# Infratemporal Fossa Glial Choristoma (Heterotopia): A Rare Presentation

**DOI:** 10.5334/jbr-btr.818

**Published:** 2016-04-11

**Authors:** Leila Aghaghazvini, Hashem Sharifian, Bahman Rasuli, Shirin Aghaghazvini, Majid Assadi

**Affiliations:** 1 Assistant Professor, Department of Radiology, Shariati Hospital, Tehran University of Medical Sciences, Tehran, Iran; 2 Assistant Professor, Department of Radiology, Amiralam Hospital, Tehran University of Medical Sciences, Tehran, Iran; 3 Department of Radiology, Advanced Diagnostic and Interventional Research Center, Tehran University of Medical Sciences, Tehran, Iran; 4 Department of Internal Medicine, Advanced Diagnostic and Interventional Research Center, Tehran University of Medical Sciences, Tehran, Iran; 5 The Persian Gulf Nuclear Medicine Research Center, Bushehr University of Medical Sciences, Bushehr 3631, Iran

**Keywords:** Glial choristoma, Heterotopia

A 7-month-old girl was referred to the head and neck department with a history of gradual painless swelling over the left side of her face for 4 months. Physical examination revealed asymmetric appearance of the face, with a 120 × 70 mm non-tender, non-mobile, and non-pulsatile prominent soft tissue mass with a smooth contour in the left parotid and periauricular spaces. Neurological examination was unremarkable.

On ultrasound examination, a solid cystic mass in the parotid space was recognized, but precise determination of the depth, margin, and extension of the mass was not possible, and complementary diagnostic studies were necessary.

MRI of the skull base and face (1) in axial (A) and sagittal (B, F) T2-weighted, coronal unenhanced (C) and post-contrast (D, E) T1-weighted sequences shows a multilobulated solid-cystic soft tissue massoccupying the infratemporal fossa with extension into the parotid and parapharyngeal spaces, without any significant enhancement. The mass was exerting pressure on adjacent structures, with no obvious intracranial and brain communication.

**Figure 1:**
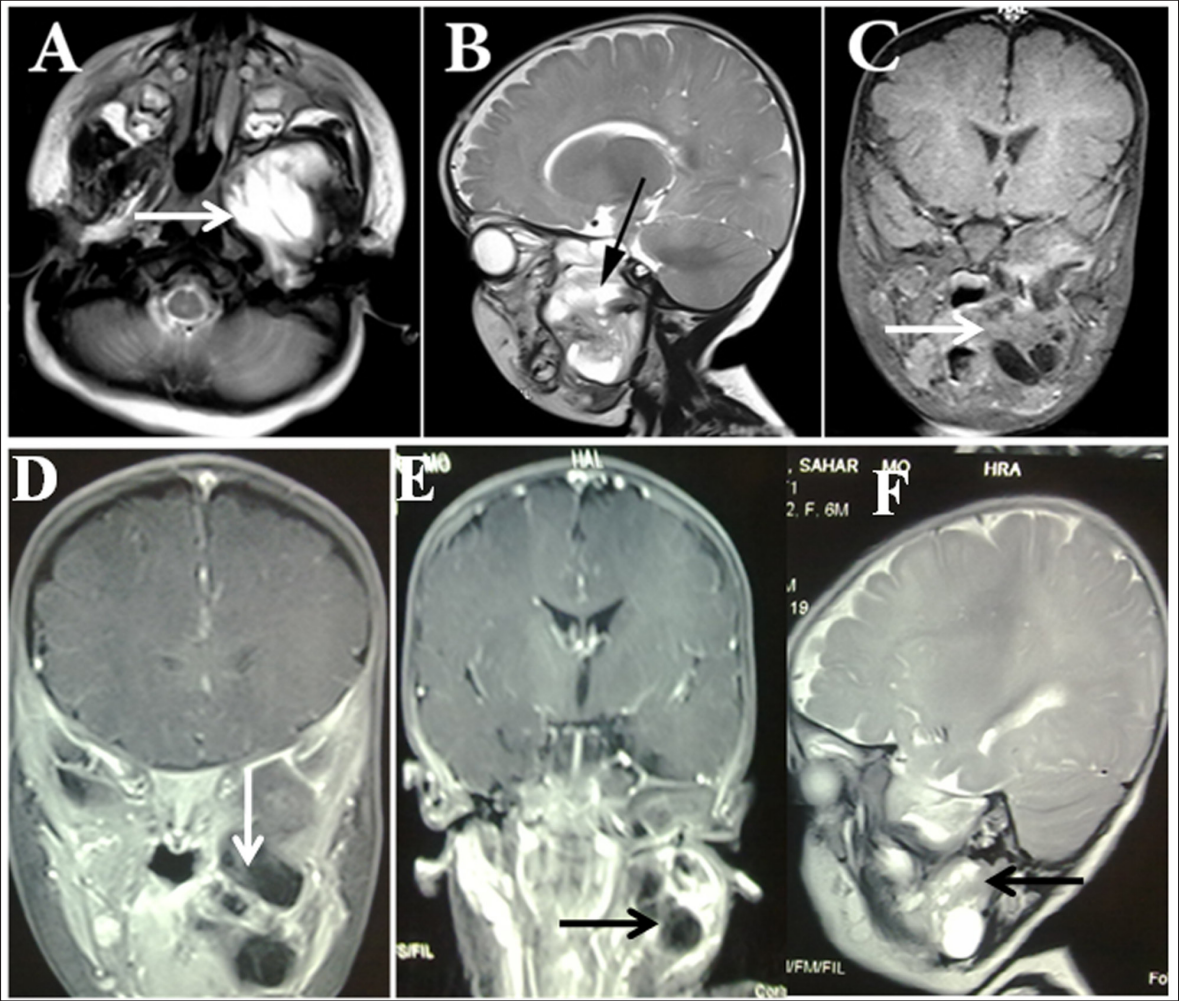


Vascular or lymphatic malformation, teratoma, encephalocele or meningocele, neoplastic lesion (such as rhabdomyosarcoma), and adenoid cystic carcinoma were considered in the differential diagnosis [1]. The patient underwent surgical resection, and the histological examination of stained sections from the infratemporal lesion showed a mass of glial tissue that consisted of several pieces of creamy-brownish tissue, consistent with glial heterotopia. On follow-up, the infant experienced unaffected development and remained asymptomatic, with no recurrence after one year.

## Comment

Extracranial glial choristoma is a rare non-teratomatous tissue that has been reported in two common sites, both distant from the neuraxis. Infratemporal glial heterotopia, also recognized as glial choristoma, is a congenital non-neoplastic migration of neuroglial tissue to an aberrant location. Glial choristoma is usually seen at birth or within the first few years of life, but can be present in any age group [1].

The most common site for these lesions is around the nasal cavity, but they may be seen in locations as varied as the scalp, head, neck, and lung [1]. Glial choristoma may assume the character of a dermoid cyst or an encephalocele due to similar embryological origins; however, it is an abnormal sequestrated tissue without a herniated element, and careful evaluation is necessary to rule out a relationship with the intracranial space [1].

CT and MRI are two important imaging modalities that should be used in the study of such masses to ascertain the anatomical site and extension of the lesion, and to rule out any communication with neural tissue. Surgical resection is the main treatment method for glial choristoma, but the lesion must be identified and distinguished from an encephalocele or other intra- and extranasal masses before surgery is planned.

Histopathologically, glial choristomas consist of neuroglial fibers and astrocytes associated with vascularized and fibrous connective tissue. The presence of plentiful neurons increases the probability of an encephalocele [1]. A few genetic diseases, such as Rubinstein-Taybi syndrome (RTS) or those involving other organs, may also feature choristomas; however, the present case had no remarkable abnormalities on physical examination [1].

Conservative surgical resection is sufficient for neuroglial choristomas, and the few reported cases of recurrence were related to inadequate resection [1]. In the present case, complete resection of the glial choristoma was achieved and no subsequent recurrence was reported during the following one year.

Our case was of interest not only because of the rarity of choristoma, but also due to its rare location in the infratemporal fossa, without intracranial communication and with normal brain tissue.

## Competing Interests

The authors declare that they have no competing interests.
